# Generation and characterization of an anti-GP73 monoclonal antibody for immunoblotting and sandwich ELISA

**DOI:** 10.7555/JBR.26.20120057

**Published:** 2012-10-13

**Authors:** Aixia Zhang, Brian Cao

**Affiliations:** a Department of Pharmacology, School of Pharmacy, Nanjing Medical University, Nanjing, Jiangsu 210029, China;; b Antibody Technology Laboratory, Van Andel Research Institute, Grand Rapids, MI 49503, USA.

**Keywords:** GP73, monoclonal antibody, Western blotting, sandwich ELISA, hepatocellular carcinoma

## Abstract

Recently, serum Golgi protein 73 (GP73) levels have been found to be elevated in patients with hepatocellular carcinoma (HCC), and GP73 has been proposed as a novel marker for HCC. However, GP73 levels in patients remain controversial due to the specificity of the anti-GP73 antibody-based enzyme linked immunosorbent assay (ELISA). Therefore, an anti-GP73 antibody with high specificity was highly demanded. In the present study, by hybridoma screening, we generated an anti-GP73 monoclonal antibody (mAb) designated as 6A2 using recombinant GP73 protein produced by prokaryotic expression. The specificity of 6A2 was evaluated by Western blotting, immunohistochemistry and immunoprecipitation. The results showed that 6A2 recognized GP73 in both native and denatured forms. In addition, we have developed a sandwich ELISA using 6A2 and GP73 polyclonal antibody generated in New Zealand white rabbits according to standard procedures, and measured the serum GP73 level of patients using this assay. Our results showed that serum GP73 levels of HCC patients were significantly higher than those of healthy controls (*P* = 0.0036). Furthermore, for the first time, GP73 serum level was found to be elevated in patients with breast cancer compared with healthy controls (*P* = 0.0172).

## INTRODUCTION

Golgi protein 73 (GP73), also termed Golgi phosphoprotein 2 (GOLPH2), is a 73-kDa type-II Golgi transmembrane glycoprotein that was originally cloned from a library derived from the liver tissue of a patient with adult giant-cell hepatitis. GP73 is constitutively expressed in cells of epithelial lineage but varies from tissue to tissue. It is abundant in the prostate, colon, breast, bronchi, thyroid gland, and central nervous system[1]. In normal human liver, GP73 is consistently expressed by biliary epithelial cells, but only minimally by hepatocytes[2]. However, abnormally high expression of GP73 was found in hepatocytes from patients with acute and chronic hepatitis, liver cirrhosis, and hepatocellular carcinoma (HCC)[3]-[5].

Despite its steady-state localization in the Golgi apparatus, the C-terminal ectodomain of GP73 is secreted into the extracelluar space by cleavage at a proprotein convertase site and present in the supernatants of several cell lines[6],[7]. Block and colleagues first reported that serum GP73 (sGP73) levels were up-regulated in patients with hepatitis B virus-related HCC[4]. Marrero et al.[8] demonstrated that the sensitivity and specificity of sGP73 for the identification of HCC were superior to those of alpha-fetoprotein (AFP), especially in early HCC. Thus, sGP73 was proposed as a novel marker for HCC diagnosis.

The method used in previous studies was immunoblotting, which is semi-quantitative, laborious, and unsuitable for routine practices. To quantitatively analyze the GP73 concentration in serum samples, two independent groups developed a sandwich enzyme linked immunosorbent assay (ELISA)[9],[10]. Although the two groups came to the same conclusion that sGP73 in HCC patients was significantly higher than that of healthy controls, the sGP73 level reported by Riener et al.[9] was approximately 100-fold higher than that reported by Gu et al.[10]. The discrepancy is most likely due to methodological differences, especially the specificity of ELISA, because some investigators have previously reported the presence of smaller GP73 antibody-reactive bands in serum by Western blotting assays[11]. Therefore, an anti-GP73 antibody with high specificity was highly demanded

Monoclonal antibodies (mAb) possess high specificity, and have been widely applied in diagnosis, treatment, and purification. In the present study, we generated an anti-GP73 mAb using recombinant GP73 protein as an antigen. The specificity of GP73 mAb was evaluated by Western blotting, immunohistochemistry (IHC) and immunoprecipitation (IP). In addition, we developed a sandwich ELISA using the GP73 mAb and GP73 polyclonal antibody (pAb). The sandwich ELISA was validated using normal serum, HCC serum and breast cancer serum samples.

## MATERIALS AND METHODS

### Reagents

TRIzol reagent and restriction endonuclease (*Bam*HI and *Hin*dIII) were purchased from Invitrogen (Carlsbad, CA, USA). All the media including Dulbecco's modified Eagle's medium (DMEM), OPTI-MEM and serum-free medium were purchased from GIBCO (Grand Island, NY, USA). The pET-28a (+) vector was obtained from Novagen (Madison, MI, USA). The HisTrap HP column and protein G column were purchased from GE Healthcare (Pittsburgh, PA, USA). Freund's complete and incomplete adjuvant, and all secondary antibodies were purchased from Sigma (St. Louis, MO, USA). Phosphatase substrate P-nitrophenylphosphate was purchased from KPL (Gaithersburg, MD, USA).

### Cell lines and animal

Human breast cancer cell line MCF-7 and prostate cancer cell line PC-3 were maintained in DMEM supplemented with 10% fetal bovine serum (FBS). All cell lines were cultured in a humidified incubator at 37°C containing 5% CO_2_.

BALB/c mice were bred and maintained at the Center of Experimental Animal of Van Andel Research Institute (USA). All studies involving animals were performed in compliance with state and institutional animal care guidelines.

### Cloning and expression of recombinant GP73

Total RNA was isolated and purified using TRIzol reagent from the HCC samples with the Institutional Review Board (IRB) approval of the Second Affiliated Hospital of Nanjing Medical University. A 1083-bp fragment of the GP73 cDNA, corresponding to the intra-Golgi protein (amino acids 41-400)[1], was amplified by polymerase chain reaction (PCR) using a primer pair: 5′-GCGGATCCCTCCAGACACGGATCATGGAGCTGGAAGGC-3′ and 5′-GCAAGCTTGGAGTGTATGATTCCGCTTTTCACGCTG-3′. The GP73 cDNA restriction fragments was inserted into the pET-28a(+) vector with a N-terminal 6′ histidine tag containing *Bam*HI and *Hin*dIII restriction sites. *E. coli* BL21 (DE3) was induced to express recombinant *GP73* (*rGP73*) with 0.4 mmol/L isopropy-β-D-thiogalactoside (IPTG) for 6 h at 37°C, and *rGP73* was purified by the HisTrap HP affinity column (GE Healthcare) according to the manufacturer's instructions.

### Generation of anti-GP73 antibodies

Mouse anti-GP73 antibodies were produced by injecting BALB/c mice intraperitoneally with purified native and sodium dodecyl sulphate (SDS)-denatured rGP73 (20 µg/mouse) suspended in Freund's complete adjuvant, followed by three additional injections in Freund's incomplete adjuvant at 3-week intervals. After the immunoreactivity against GP73 was validated, the final boost was given without adjuvant. Four days later, spleen cells were isolated from the sacrificed mice and then were fused with the OUR-1 myeloma cells using standard techniques, and hybridomas were generated by the method described previously[12]. To screen for positive hybridoma clones, we coated 96-well plates with 2.0 mg/L of rGP73 in a coating buffer (0.2 mol/L Na_2_CO_3_/NaHCO_3_, pH 9.6) at 4°C overnight. After washing twice with washing buffer (PBS with 0.05% Tween-20, PBST), the plates were then blocked with PBS containing 1% bovine serum albumin (BSA) overnight at 4°C. Fifty µl hybridoma supernatant was added to the wells and incubated for 1.5 h at room temperature (RT). Plates were washed twice and an alkaline phosphatase (AP)-conjugated goat anti-mouse IgG in a 1:2,000 dilution was added and incubated for 1.5 h at RT. After washing four times, P-nitrophenylphosphate, a phosphatase substrate, was added and incubated for 30 min, and then absorbance was measured at 405 nm. The hybridoma clones with strong reactivity with rGP73 were re-cloned twice by limited dilution, and their reactivity was re-confirmed by ELISA.

Subcloned hybridoma cells were cultured in the OPTI-MEM medium containing 10% FBS, weaned gradually to serum-free medium, and then transferred to the Bioreactor (INTEGRA Biosciences AG, CH-7000 Chur, Switzerland). The anti-GP73 mAb was purified from the culture supernatants by affinity chromatography using a protein-G column. GP73 pAb were produced in New Zealand white rabbits according to standard procedures[13]. Rabbit immune sera were purified by a protein-G column

### Western blotting assays

To screen for antibodies used for Western blotting assays, 20 µg of rGP73 was electrophoresed in a two dimensional SDS-polyacrylamide gel electrophoresis (2D SDS-PAGE), and transferred onto a nitrocellulose membrane. The membrane was blocked with 5% of milk for 2 h at room temperature. A multi-channel apparatus (Bio-Rad, Irvine, CA, USA) was used to probe the membrane with 650 µL supernatants of each original ELISA-positive clone at 4°C overnight. The blotting assays were detected using a horseradish peroxidase (HRP) conjugated goat anti-mouse secondary antibody and an Enhanced Chemiluminescence Kit (Pierce, Rockford, IL, USA). For the identification of purified GP73 monoclonal antibody, rGP73 protein (0.1 µg per lane) was electrophoresed in SDS-PAGE and transferred to a nitrocellulose membrane. The membrane was probed with pre-immune mouse serum, anti-GP73 monoclonal antiobody and final booster mouse serum.

### Immunoprecipitations

Cell lysate was prepared from breast cancer cell line MCF-7 and prostate cancer cell line PC-3 using a RIPA buffer (20 mmol/L Tris-HCl, pH 7.4; 1% NP-40; 150 mmol/L NaCl, and protease inhibitors) (Roche, Indianapolis, IN, USA). Three hundred µl cell lysate was immunoprecipitated overnight with 5 µg anti-GP73 mAb coupled to protein G beads. Afterward, beads were washed twice with PBST, and 20 µl each sample was separated by SDS-PAGE. For Western blotting, anti-GP73 polyclonal antibody was used as primary antibodies (1:2,000), and a HRP-labeled goat anti-rabbit IgG (1:2,000) as secondary antibodies.

### Immunohistochemistry

Formalin-fixed and paraffin-embedded tissue blocks of hyperplasic prostate and HCC were obtained from the First Affiliated Hospital of Nanjing Medical University with the approval by the local institutional review board. For IHC staining, sections (4 µm) from tissue blocks were mounted on positively charged glass slides, and then baked, deparaffinized and rehydrated. Antigen retrieval was achieved by heating slides in a citrate buffer (10 mmol/L, pH 6.0) for 20 min in a pressure cooker. The sections were incubated overnight at 4°C with the GP73 monoclonal antibody in a 1:2,000 dilution. After washing with PBS, the sections were incubated with anti-mouse HRP-polymer (Maixin. Bio, Fuzhou, China) at room temperature for 30 min. Localization of the anti-GP73 monoclonal antibody was visualized with 3,3′-diaminobenzidine (Vector Laboratories, Burlingame, CA, USA) as a chromogen.

### GP73 sandwich ELISA

GP73 was measured using a double-antibody sandwich ELISA in a 100-µL reaction system with the mouse anti-GP73 monoclonal antibody as the capture antibody and rabbit anti-GP73 polyclonal antibody as the tracer antibody. The AP-conjugated anti-rabbit IgG was used as the detection antibody and rGP73 protein as the standard. The concentrations of all antibodies and rGP73 protein were determined by BCA assays (Pierce, Rockford, IL, USA).

For sandwich ELISA, the 96-well plate was coated with 100 µL the GP73 monoclonal antibody at a concentration of 1 mg/L in a coating buffer overnight at 4°C. The plate was washed and blocked with 1% milk (20 mmol/L Tris, 0.1 mol/L NaCl, pH 8.0) at 4°C overnight. The blocking solution was removed and the rGP73 standards were added using 0.25 µg/L as the initial concentration followed by serial dilutions. After incubation at 37°C for 1.5 h, the plate was washed and the rabbit anti-GP73 polyclonal antibody (1:1,000) was added and incubated for 1.5 h. After washing, the AP-conjugated anti-rabbit IgG (1:2,000) was added and incubated for an additional 1.5 h at room temperature. The AP substrate was added and incubated for 30 min at room temperature The absorbance was measured at 405 nm.

**Fig. 1:**
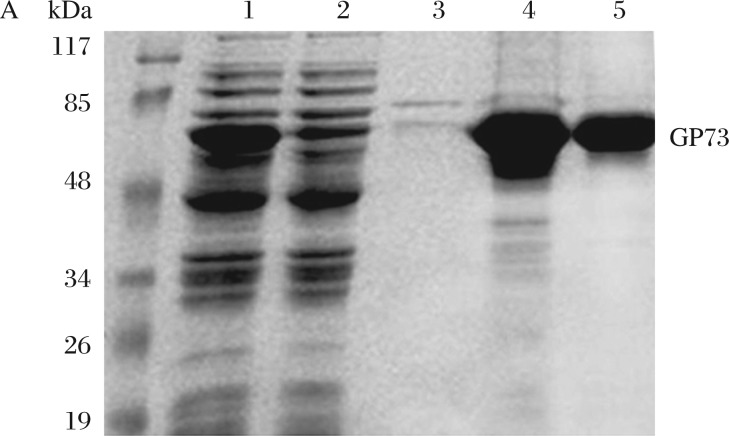
Purification and identification of rGP73 protein. A: SDS-PAGE of rGP73 protein purified by Ni affinity chromatography. Lane 1: crude lysate, lane 2: flow through, lane 3-5: eluate washed by 0.1, 0.3, and 0.5 mol/L imidazole, respectively. B: Mass spectrum analysis of rGP73 protein. Database search by the MASCOT program verified the identification of GP73. Matched peptides are shown in gray, with peptide sequence coverage of 75%.

**Fig. 2:**
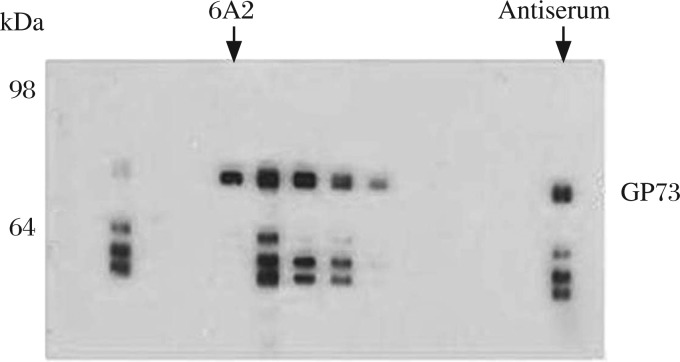
Screening of GP73-reactive fusion hybridoma clones. A total of 20 µg GP73 proteins was separated by 2D SDS-PAGE, and transferred onto a nitrocellulose membrane, which was probed with 650 µL supernatants of each original ELISA-positive clone. The final boost mouse serum was used as the positive control.

**Fig. 3:**
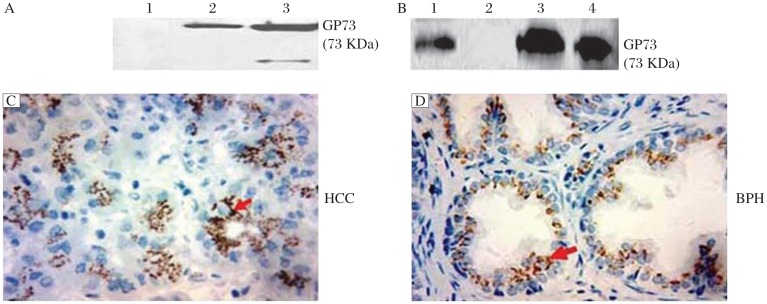
Characterization of 6A2. A: The specificity of 6A2 was confirmed by Western blotting assays. Lane 1: pre-immune mouse serum, lane 2: purified 6A2, lane 3: final boost mouse serum. B: Immunoprecipitation of 6A2. Lane 1: a positive control (0.2 µg rGP73 protein), lane 2: a negative control (6A2 + RIPA buffer), lane 3: PC-3 cell lysate, lane 4: MCF-7 cell lysate. IHC staining using 6A2 on sections of HCC (C) and BPH (D) tissue (×400). GP73 staining was mainly detected in the perinuclear region of HCC cells and in the luminal side of prostatic gland epithelia (BPH).

Fifty-six serum samples (11 HCC, 16 breast cancer and 30 healthy controls) were collected from Shanghai Tongji Hospital. The consent form and study design were approved by the institutional review board of the Shanghai Tongji Hospital. Microtiter plates were coated with 1 mg/L the GP73 monocloanl antibody as described above. After blocking with 1% milk overnight, serially diluted rGP73 standard and the serum samples (1:2 in 1% of milk) were added to their respective wells. All standard protein and samples were analyzed in duplicate. The following steps were completed according to the ELISA assay protocol described above.

### Statistical analysis

All data were expressed as mean±SD. SPSS 16.0 (SPSS Inc., Chicago, IL, USA) was used for statistical analysis. Statistical analysis among multiple groups were performed with one-way ANOVA followed by Scheffe's post hoc test. Comparisons between two groups were made with a two-tailed Student's *t*-test. A *P*-value < 0.05 was considered statistically significant.

## RESULTS

### Expression and purification of rGP73

To obtain rGP73 protein, we amplified the GP73 fragment from HCC frozen tissues by RT-PCR and inserted it into pET-28a (+). The rGP73 protein was successfully expressed in BL21 (DE3) strain, and then was purified by affinity chromatography (***Fig. 1A***). To confirm whether the only 73k-Da band on the gel was indeed GP73, we performed a mass spectrometry analysis. The sequences searched from the database by the MASCOT program confirmed that the target protein was indeed GP73 (***Fig. 1B***).

### Generation of anti-GP73 antibody

To prepare polyclonal and monoclonal antibodies against GP73, we immunized rabbits and BALB/c mice using purified rGP73 as antigen, respectively. After three immunizations, the antibody titer in rabbits and mice reached up to 1:128,000. After the final injection, the spleen cells of mouse with the highest titer were fused with myeloma cells. Eight clones with the highest optical density (OD) values in the capture ELISA were chosen and expanded.

In screening positive clones for Western blotting assays, purified rGP73 protein was separated by SDS-PAGE and transferred to membranes before probing with the supernatants of ELISA-positive clones. Among the 8 clones, 6A2 was chosen and subcloned due to its highest signal-to background ratio against rGP73 (***Fig. 2***).

### Characterization of monoclonal antibodies against GP73

After purification, the specificity of the GP73 mAb 6A2 was tested by western blotting assays with rGP73 protein. As shown in ***Fig. 3A***, the band around 73 kDa was recognized by both 6A2 and the final boost serum, but not by the pre-immune serum. To investigate if 6A2 could recognize the native GP73 protein, we performed IP with PC-3 and MCF-7 cell lysate, both of which express GP73 positively[14]. As shown in ***Fig. 3B***, the endogenous GP73 protein was pulled down by 6A2 from the lysates of PC-3 and MCF-7. This result demonstrated that 6A2 could recognize the native GP73 protein.

GP73 is a type II transmembrane protein localized in the cis and medial-Golgi cisternae. Immunohistochemically, GP73 was rarely detected in normal hepatocytes but was significantly expressed in HCC cells. Recently, up-regulation of GP73 has been reported in benign and malignant prostatic lesions[14],[15]. To further characterize 6A2, we performed IHC staining with 6A2 on tissue sections of HCC and benign prostatic hyperplasia (BPH). As shown in ***Fig. 3C***, in the HCC tissue, a distinct semi-granular dot-like staining pattern was localized perinuclearly in HCC cells, whereas the rest of the cytoplasm was non-stained. In BPH, the predominant juxtanuclear staining pattern was mainly localized on the luminal side of the prostatic gland epithelia (***Fig. 3D***). These staining patterns are in agreement with previous studies on the intracytoplasmic localization of GP73 in HCC and prostate lesions, and demonstrated the specificity of 6A2.

**Fig. 4:**
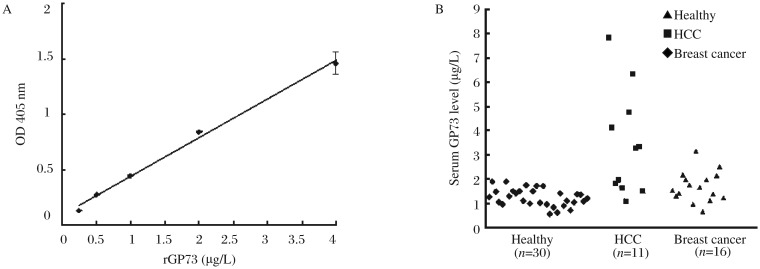
Establishment and validation of GP73 sandwich ELISA in clinical samples. A: Standard curves of GP73 sandwich ELISA. A 96-well plate was coated with 1 mg/L 6A2 at 4°C overnight. After blocking, rGP73 standards were added using 0.25 µg/L as the initial concentration followed by serial dilutions. After incubation at 37°C for 1.5 h, the plate was washed and incubated with rabbit anti-GP73 polyclonal antibodies (1:1,000) for 1.5 h. AP-conjugated secondary antibody was used to detect the reactivity. Absorbance was measured at 405 nm. B: Scatter plots showing serum GP73 levels in healthy controls, HCC and breast cancer patients.

**Table 1:** Serum GP73 level (µg/L) in clinical samples

Group	*n*	Serum level (µg/L)	*P*
Healthy	30	1.227±0.359	0.0036*
HCC	11	3.44±2.178	
Breast cancer	16	1.665±0.6234	0.0172^#^

*P healthy vs HCC; ^#^*P* healthy vs breast cancer.

### Validation of GP73 sandwich ELISA in clinical samples

Accumulating evidence suggested that GP73 could be a potential marker for the diagnosis of HCC, and thus sandwich ELISA assays have been developed for the quantification of sGP73[9],[10]. However, notable differences in sGP73 levels existed in those reports due to the specificity of ELISA. We established a double antibody sandwich ELISA using a combination of 6A2 as the capture antibody and the rabbit GP73 antibody as the tracer antibody. A typical standard curve for the quantification of GP73 is shown in ***Fig. 4A***. At a range of 0.25-4.0 µg/L GP73, there was a good liner relationship between protein concentrations and absorbance. To validate the use of GP73 sandwich ELISA in clinical serum samples, we examined GP73 levels in patients with HCC (*n* = 11), patients with breast cancer (*n* = 16), and healthy controls (*n* = 30). The results showed that sGP73 levels of HCC patients were in the range of 1.087-7.848 µg/L (median = 3.441 µg/L) (***Fig. 4B***), which was significantly higher than that of healthy controls (median = 1.227 µg/L, *P* = 0.0036). In addition, a significant difference in GP73 levels was found between breast cancer patients and healthy controls (*P* = 0.0172)(***Table 1***).

## DISCUSSION

GP73 was originally described as a resident type II Golgi transmembrane protein expressed primarily in epithelial cells of human tissues[1]. GP73 expression was barely found in normal hepatocytes but markedly up-regulated in hepatocytes from patients with viral and non-viral liver diseases, and especially HCC[2]-[5]. Since Block et al.[4] first demonstrated that GP73 was upregulated in sera of patients with hepatitis B virus-related HCC, more and more research showed that the sensitivity and specificity of sGP73 for HCC were superior to those of AFP, especially in early HCC[5],[8],[16],[17]. With these encouraging reports, sGP73 is believed to be a valuable marker for HCC diagnosis. The quantification of sGP73 levels using a sandwich ELISA has been reported by the investigators from different groups[9],[10]. Although there was a high concordance in results and conclusions between these groups, sGP73 levels reported by Riener et al.[9] were about 100 times higher than those reported by Gu and colleagues[10]. The great discrepancy raises questions regarding the specificity of ELISA, because Riener et al. reported that the median serum GP73 concentration of normal subjects was 4 mg/L, which is well within the range of many classical plasma proteins[18], and other investigators have previously reported the presence of smaller GP73 antibody-reactive bands in Western blotting assays of serum[11]. This issue could best be resolved by parallel comparisons of Western blotting assays and ELISA in the same samples. This encourages us to generate an anti-GP73 monoclonal antibody of high specificity, which possesses a universal application in immunoblotting and ELISA.

An appropriate screening is critical to hybridoma production. The screening is selected on the basis of the characteristics of the antigen and the use of the antibody. To generate an anti-GP73 monoclonal antibody which possesses a universal application in immunoblotting and ELISA, we immunized mice with GP73 in both native and denatured forms. After three immunization injections, the mouse with the highest antibody titer was given a final boost, and then its spleen cells were fused with myeloma cells. To screen antibody that will work in Western blotting assays, a total of 8 hybridomas having the strongest reactivity with GP73 by ELISA were tested whether they work in Western-blotting assays. Among these clones, 6A2 was chosen because it showed the highest signal-to background ratio (***Fig. 2***). The Western blotting assays demonstrated high specificity of 6A2 monoclonal antibodies against denatured GP73 (***Fig. 3A***). To investigate if 6A2 could recognize native GP73, we performed immunoprecipitation assays with the lysate of GP73 positive cell lines. The result demonstrated that 6A2 specifically pulled down native GP73 (***Fig. 3B***), and it also works for the sandwich ELISA.

It has been reported that GP73 expression was upregulated in HCC cells and the glandular epithelium of prostate lesions[14],[19]. We performed IHC staining on HCC and BPH tissue sections with 6A2. Our result showed that the staining patterns are consistent with other reports on the intracytoplasmic localization of GP73 in HCC and prostate lesions (***Fig. 3C*** and ***3D***), which further confirmed the specificity of 6A2.

We set up a sandwich ELISA using a combination of 6A2 and GP73 polyclonal antibody. Three independent standard curves were constructed at different times, demonstrating that the system is reliable and reproducible ***Fig. 4***. The concentration of detection limit of this detection system was as low as 0.25 µg/L, indicating its high sensitivity. We tested sGP73 levels in patients with HCC and healthy controls. Our results showed that serum GP73 levels of HCC patients were significantly higher than those of healthy controls (*P* = 0.0036), which is consistent with previous reports. The sGP73 concentration in this study was about 50-fold lower than that reported by Gu et al.[10]. This discrepancy might be due to the specificity and affinity of GP73 antibodies, which are critical to ELISA sensitivity. The antibody titer of 6A2 was 1:1,280,000.

In addition to HCC, GP73 was constitutively expressed in the epithelia of breast tissues and was significantly upregulated in invasive breast cancer[1],[14]. We questioned if GP73 was significantly upregulated in the serum of patients with breast cancer. Surprisingly, there was a significant difference in sGP73 levels between breast cancer patients and healthy controls (1.665±0.6234 µg/L vs 1.227±0.359 µg/L, *P* = 0.0172) (***Table 1***). To our knowledge, this is the first report to show elevated expression of sGP73 in breast cancer patients. However, our study was based on a relatively small number of HCC and breast cancer cases, with some cases that showed recurrence after surgery and/or chemotherapy. The relevance of sGP73 level and the prognosis of HCC or breast cancer needs to be further explored in a larger number of patients.

In summary, we generated anti-GP73 mAB for immunoblotting and sandwich ELISA. This study points to the potential of anti-GP73 mAB as an optimal antibody in evaluating sGP73 levels by Western-blotting assays and ELISA clinically.
